# Surface Excess Energy as a Unifying Thermodynamic Framework for Active Diffusion

**DOI:** 10.1021/acs.jpcb.5c07096

**Published:** 2026-01-23

**Authors:** Andrés Arango-Restrepo, J. Miguel Rubi

**Affiliations:** † Departament de Física de la Matèria Condensada, Facultad de Fisica, 16724 Universitat de Barcelona, Barcelona 08028, Spain; ‡ Institut de Nanociencia i Nanotecnologia, Universitat de Barcelona, Barcelona 08028, Spain

## Abstract

Directed motion of particles is typically explained by phoretic mechanisms arising under externally imposed chemical, electric, or thermal gradients. In contrast, chemical reactions can enhance particle diffusion even in the absence of such external gradients. We refer to this increase as active diffusivity, often attributed to self-diffusiophoresis or self-electrophoresis, although these mechanisms alone do not fully account for experimental observations. Here, we investigate active diffusivity in catalytic Janus particles immersed in reactive media without imposed gradients. We show that interfacial reactions generate excess surface energy and sustained interfacial stresses that supplement thermal energy, enabling diffusion beyond the classical thermal limit. We consistently quantify this contribution using both dissipative and nondissipative approaches, assuming that the aqueous bath remains near equilibrium. Our framework reproduces experimentally observed trends in diffusivity versus activity, including the nonmonotonic behaviors reported in some systems, and agrees with data for nanometric Janus particles catalyzing charged substrates as well as vesicles with membrane-embedded enzymes driven by ATP hydrolysis. These results demonstrate that chemical reactions can induce and sustain surface-tension gradients and surface excess energy, providing design principles for tuning mobility in synthetic active matter.

## Graphical abstract

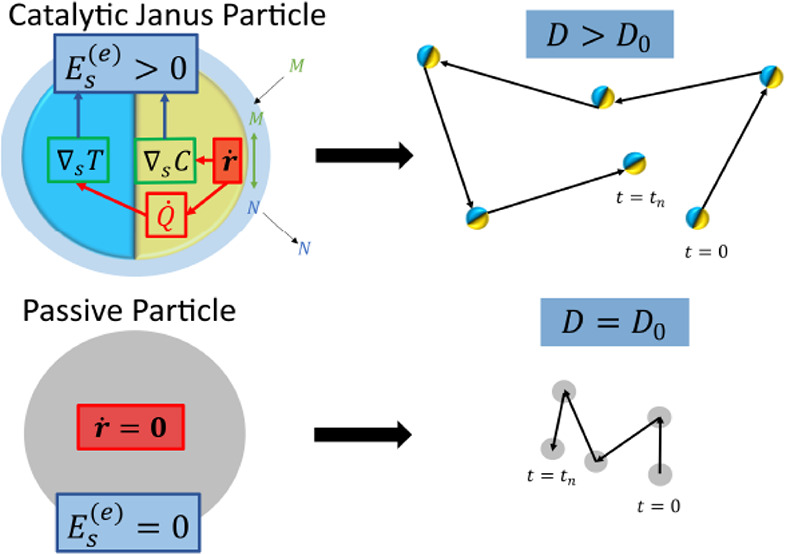

## Introduction

Particle self-propulsion is most commonly explained by phoretic mechanisms driven by external forces or imposed gradients.^1^ Stochastic thermodynamics offers a theoretical framework for understanding the dynamics of hot Brownian swimmers.^2,3^ Nonequilibrium thermodynamics combined with hydrodynamics shows that thermophoretic mobilities depend sensitively on boundary conditions,^4,5^ consistent with Faxén-type relations connecting surface-tension gradients to hydrodynamic forces.^6^ Related studies emphasize how temperature gradients couple thermophoretic forces and flow fields.^7,8^ These works primarily address propulsion under external gradients rather than self-generated gradients in chemically reactive systems. Models of chemically induced phoresis have been proposed,^9,10^ but they have largely focused on directed motion and phoretic velocities, leaving active diffusivity less explored. A recent study showed that applying a constant external force to randomly oriented active particles enhances diffusivity, producing an essentially constant scaling with activity.^11^ Since this behavior is not universal, we aim to overcome these limitations. The enhanced mobility of chemically active nano and microparticles has been widely attributed to self-phoretic mechanisms, particularly self-diffusiophoresis, where solute concentration gradients, generated by surface chemical reactions, induce slip flows along the particle interface, resulting in propulsion.^12,13^ This framework has explained a variety of experimental observations, especially in asymmetric systems such as catalytic Janus particles.^14,15^ Nonetheless, self-diffusiophoresis alone does not fully capture the breadth of experimentally observed diffusivity behaviors, especially regarding their nonlinear dependence on reaction rates.^16,17^ Self-electrophoresis has been introduced as a complementary mechanism^18,19^ yet a unified understanding remains lacking.

A thermodynamically grounded yet often underappreciated mechanism is self-thermophoresis, arising from temperature gradients produced by exothermic surface reactions^18,20^ While extensively studied in externally forced systems.^21,22^ Its contribution as a self-generated driving force in active particles is less explored. Moreover, existing models tend to treat diffusiophoresis, electrophoresis, and thermophoresis in isolation, overlooking the possible couplings among them and their collective impact on particle dynamics.

At a fundamental level, self-phoretic motion arises from thermodynamic gradients, such as concentration, temperature, or electric potential of the solute, generated by chemical reactions on the surface of the particle.^18,23^ These gradients induce slip flows that drive motion but also modify the surface excess energy, defined as the energy stored at the interface due to local deviations from ideality. Importantly, the formation and maintenance of such gradients lead to irreversible processes that generate entropy at the interface, sustaining a nonequilibrium steady state.^24^ This entropy production reflects ongoing energy dissipation, which plays a direct role in enhancing particle mobility.

This perspective shifts the focus from purely mechanical descriptions of propulsion to a thermodynamic interpretation, where surface tension gradients, energy fluxes, and entropy generation collectively determine the mobility of active particles. Crucially, these interfacial thermodynamic variables are accessible through measurable quantities and can be used to develop predictive, quantitative models of active diffusivity. As we show in this work, incorporating both nondissipative and dissipative contributions to the surface excess energy provides a comprehensive understanding of how interfacial processes govern enhanced particle transport.

In this work, we develop a thermodynamic framework to explain the enhanced diffusivity of Janus catalytic particles with respect to passive particles due to self-propulsion, as observed in experiments, through explicit analysis of the nonequilibrium processes taking place in the particle–fluid interface. Our goal is to unify the main self-phoretic mechanisms (diffusiophoresis, electrophoresis, and thermophoresis) through their connection to excess surface energy. We calculate the excess surface energy generated by catalytic reactions using two complementary approaches: a nondissipative one, which captures how reaction-induced gradients modify the interfacial energy, and a dissipative one, which considers entropy generation and energy dissipation. Both perspectives reveal how catalytic activity and thermal energy enhance diffusion beyond the classical thermal limit, considering that the vast aqueous media is at thermal equilibrium. From the resulting expression for diffusivity, we recover the experimentally observed nonmonotonic dependence of diffusivity on reaction rate. To validate the framework, we analyze two experimentally studied systems: nanometric Janus particles using charged salts as substrates,^20^ and phospholipid vesicles with integrated enzymes that hydrolyze ATP.^25^ Our findings highlight surface tension gradients and interfacial entropy production, through excess surface energy, as central regulators of active diffusivity and provide a solid physical basis for the design of synthetic active matter.

The paper is organized as follows. Section II presents the system and describes the theoretical framework of active diffusivity, along with the estimation of excess surface energy from dissipative and nondissipative perspectives. Section III presents the model based on conservation equations. In Section IV, we derive analytical expressions for surface energy excess using both approaches. Section V contains our results, including a comparison with experimental data, and highlights the nonlinear and nonmonotonic dependence of active diffusivity on activity (reaction rate). Finally, Section VI summarizes our main conclusions.

## Theory

### System

We consider a catalytic Janus particle where a chemical reaction on one side of the surface generates asymmetric concentration and temperature fields. On the surface of the particle, an irreversible chemical reaction takes place, which converts a substrate *M* into products *N*. This process occurs in a medium without external flow and interparticle interactions. At the interface (*i*) between the particle (*p*) and the surrounding fluid (*b*), all chemical species are present. Figure 1 depicts a spherical catalytic Janus particle and its surrounding environment. The active diffusion is primarily governed by the surface excess energy, $E_{s}^{\left(e\right)}$ , which results from surface-tangential concentration and temperature gradients (∇_*S*_ *C*_*M*_, ∇_*S*_ *C*_*N*_, ∇_*S*_ *T*), the electrostatic potential ψ, and surface entropy *S*. These variations originate from the reaction rate *ṙ* and the heat generated by the reaction, *ṙ*Δ*H*_*r*_, within the catalytic region of the particle.

**Figure 1:**
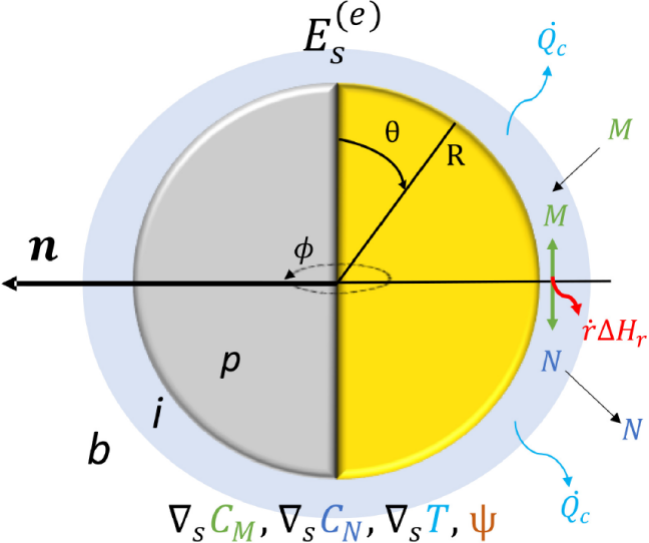
Illustration of a catalytic Janus particle undergoing a first-order reaction at its interface, where substrate *M* is converted into product *N* at rate *ṙ* producing heat *ṙ*Δ*H*_*r*_, and thereby inducing an excess surface energy $E_{s}^{\left(e\right)}$ . Here, the particle is depicted with an orientation **n**, extending from the catalytic (golden color) to the noncatalytic side (gray color). The interface region *i* is located between the particle *p* and the surrounding fluid *b*. From the interface, the heat flux *Q̇*_*c*_ is transferred to the fluid, while *M* is being absorbed from the fluid. The surface of the particle is parametrized by the polar θ and azimuthal ϕ angles. ∇_*S*_ represents the surface-tangential gradient operator.

### Active Diffusivity

We consider a spherical particle of mass *m* and moment of inertia *I*, whose orientation is described by a unit vector **n**. The inertial Langevin equations governing the translational velocity **v**(*t*) and the angular velocity **ω**(*t*) are:

1 $$m\mathrm{v̇}=-\xi_{t}v+F_{\mathrm{ph}}+F_{t}\left(t\right)$$

2 $$I\mathrm{\omega ̇}=-\xi_{r}\omega +T_{\mathrm{ph}}+T_{r}\left(t\right)$$

where ξ_*t*_ and ξ_*r*_ are the translational and rotational friction coefficients, respectively, **F**_*ph*_ = ξ_*t*_ *b* **n** is the phoretic (active) force with **n** the orientation vector and *b* to be determined, whereas **T**_*ph*_ is the phoretic (active) torque fulfilling **T**_**ph**_⊗**n** = (**I**-**nn**)· ∇_**n**_ψ, with $\psi =E_{s}^{\left(e\right)}{\left(1-n\cdot m\right)}^{2}$ the orientation potential and **m** the external field unitary director vector.^26^ The random force for translational velocities **F**_*t*_ and the random torque for angular velocity **T**_*r*_ are Gaussian white noises that fulfill the fluctuation–dissipation theorem: ⟨**F**_*t*_ *(t)* **F**_*t*_(*t*′)⟩ = 6ξ_*t*_ *k*_*B*_ *T* ^(*b*)^ **I**δ­(*t* - *t*′) and ⟨**T**_*r*_(*t*)**T**_*r*_(*t*′)⟩ = 6 ξ_*r*_ *k*_*B*_ *T* ^(*b*)^ **I**δ­(*t* - *t*′) with *T* ^(*b*)^ the bulk/bath temperature.

The orientation vector **n** evolves according to:

3 ṅ=ω×n

in which ||**n**|| = 1 for all times. In the absence of external fields or gradients that could align the particle,^24^ the phoretic torque is negligible compared to the rotational noise, thus **n** is random, so its correlation for long times is ⟨**n**(*t*)**n**(*t*′)⟩ ≈ **I**δ­(*t* - *t*′) (see Supporting Information A).

We can then see that the random phoretic force fulfills the fluctuation–dissipation theorem ⟨**F**_*ph*_(*t*) **F**_*ph*_(*t*′)⟩ = 6 ξ_*t*_ *B* **I**δ­(*t*-*t*′), with *B* being the phoretic energy of the particle-fluid interphase. Solving Eq. ([Disp-formula eq1]) (see Supporting Information B) for long times, we obtain the mean kinetic energy of the particle as a function of the thermal energy of the solvent and the resulting magnitude of the excess surface energy $\left|E_{s}^{\left(e\right)}\right|$ on the particle due to the reaction

4 $${\frac{m}{2}\langle v(t)}^{2}\rangle_{t\to \infty }=\left|E_{s}^{\left(e\right)}\right|+k_{B}T^{\left(b\right)}$$

Notice that in equilibrium, the fluctuation–dissipation theorem (FDT) ensures that fluctuations and dissipation are balanced, with diffusivity determined solely by the thermal energy of the solvent via the equipartition theorem. However, for active particles, additional energy—originating from chemical reactions or surface activity—injects fluctuations beyond those allowed by thermal equilibrium. This leads to a violation of the FDT: the particle experiences enhanced movement and mean-squared velocity without a corresponding change in friction. Mechanically, this means the diffusivity reflects not just thermal fluctuations but also excess surface energy, effectively raising the energy available per degree of freedom and resulting in faster motion even in the absence of external forces.

Considering that the mean displacement of the particle is given by $\Delta x\left(t\right)=\int_{0}^{t}v\left(t\right)dt$ with *v*(*t*) given in Eq.(S.3),^27^ we can obtain the mean square displacement ⟨Δ*x* ^2^⟩(*t*) for long times from ([Disp-formula eq1])^24^

5 $${\langle \Delta x(t)}^{2}\rangle_{t\gg t_{0}}=2\left(\frac{k_{B}T^{\left(b\right)}}{\xi_{t}}+\frac{\left|E_{s}^{\left(e\right)}\right|}{\xi_{t}}\right)t$$

with *t*_0_ the microscopic momentum-relaxation (inertial) time. Therefore, from ([Disp-formula eq5]), the active diffusivity is given by

6 $$D=D_{0}\left(1+\frac{\left|E_{s}^{\left(e\right)}\right|}{k_{B}T^{\left(b\right)}}\right)$$

where *D*_0_ is the diffusivity of the particle in the absence of reaction.

### Excess Surface Energy

The surface excess energy of an active particle arises from local mass and heat fluxes from a surface reaction that also generate entropy at the interface. The chemical reaction then drives energy input at the surface, increasing fluctuations and enhancing the particle’s mobility. As a result, the surface becomes a source of nonequilibrium energy, linking fluxes (or gradients) and entropy production directly to the particle’s effective diffusivity. Therefore, in this subsection we will define the surface excess energy from a nondissipative (surface tension gradient) and dissipative (entropy production) perspectives.

In Figure 2, we present a diagram summarizing how excess surface energy mediates the conversion of chemical energy into particle dynamics, from both a dissipative and nondissipative perspective. From the nondissipative perspective, chemical reactions on the catalytic side of a Janus particle generate interfacial concentration and temperature gradients that modify the surface tension, chemical potential, and enthalpy of the interface. These changes represent the energy cost of breaking the homogeneous distribution of interfacial energy, maintaining surface gradients, and define an excess surface energy that adds to the thermal energy of the medium experienced by the particle, thus improving its effective (active) diffusivity compared to its passive counterpart driven solely by thermal fluctuations. From a dissipative perspective, the same chemical activity induces flows and gradients that lead to the production of entropy at the interface, resulting in more negative free energy. This excess surface energy must be dissipated and generates the thermodynamic driving force for phoretic motion, directly linking entropy production with directed transport.

**Figure 2:**
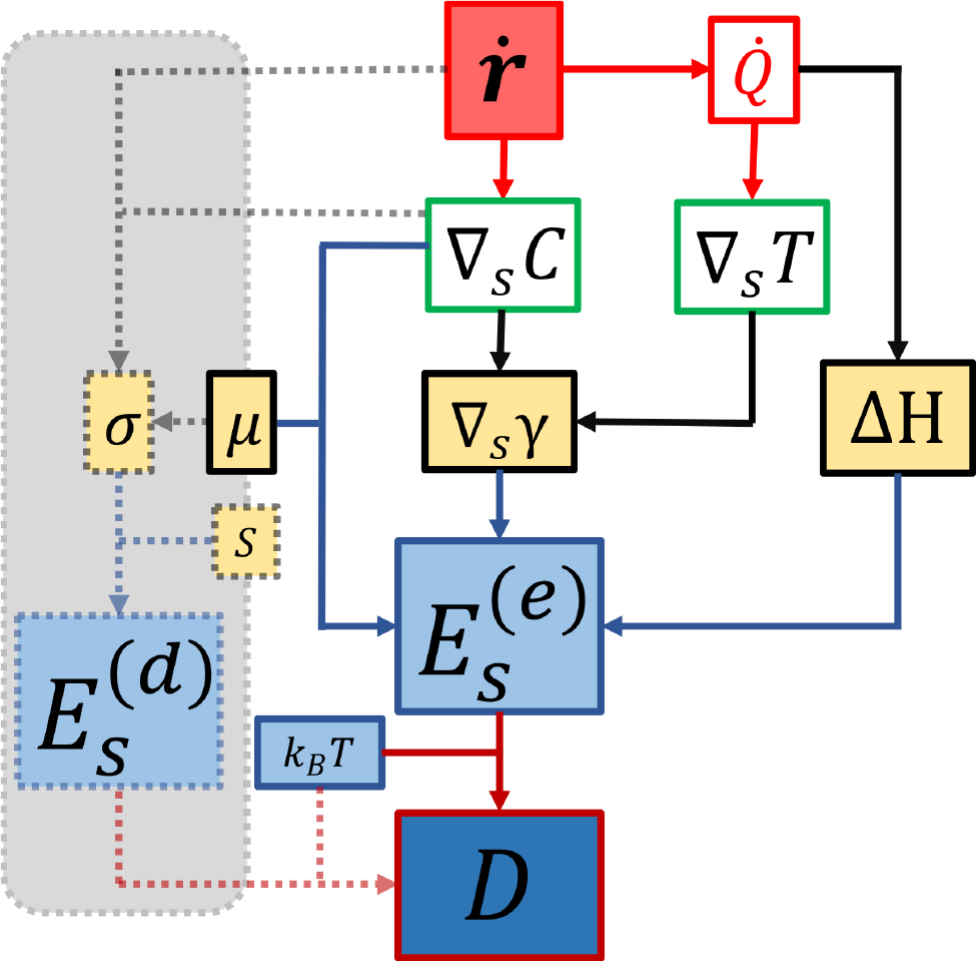
Schematic summarizing the role of surface excess energy in active systems. Continuous lines indicate the nondissipative pathway, in which a chemical reaction rate *ṙ* generates surface concentration gradients ∇_*s*_ *C* and releases heat *Q̇*, leading to surface temperature gradients ∇_*s*_ *T*. These gradients induce variations in interfacial energy ∇_*s*_γ which, together with changes in chemical potential μ and enthalpy Δ*H*, contribute to the surface excess energy $E_{s}^{\left(e\right)}$ , thereby enhancing the active diffusivity. Dashed lines denote the dissipative pathway, where entropy production—arising from chemical reactions and interfacial gradients—irreversibly reduces the available free energy, quantified by the dissipative surface excess energy $E_{s}^{\left(d\right)}$ , and acts as the thermodynamic driving mechanism underlying the active diffusivity.

#### Nondissipative Approach

To determine the excess surface energy, we analyze an infinitesimal local change in the interfacial energy, *U* = *Au*, where *A* denotes the surface area of the particle,

7 $$dU=\delta Q+\underset{i}{\sum }\mu_{i}dN_{i}+\gamma dA$$

Here δ*Q* represents the reversible heat exchanged between the interface and the fluid, including the heat released by a chemical reaction taking place at *dA*, μ_*i*_ denotes the chemical potential of component *i*, and *N*_*i*_ is the number of moles of component *i* at *dA*.

Defining the specific surface excess energy $e_{s}^{\left(e\right)}$ as the difference between the internal energy per area *u* and the surface tension γ: $e_{s}^{\left(e\right)}=u-\gamma$ , and rewriting the energy balance ([Disp-formula eq7])), we obtain the differential variation of the specific surface energy excess

8 $$de_{s}^{\left(e\right)}=\delta q+\underset{i}{\sum }\mu_{i}dC_{i}-d\gamma$$

with *q* = *Q*/*A* and *C*_*i*_ = *N*_*i*_/*A*. For a nonreactive system composed of a pure fluid, we recover the well-known result $e_{s}^{\left(e\right)}=-T\partial \gamma /\partial T$ , as previously reported in ref.^28^

Focusing on the heat and work required to increase the excess surface energy in a reactive system, we consider the following:

1) The surface heat variation is determined by the reversible heat locally produced (or consumed) by an exothermic (or endothermic) reaction, given approximately by δ*q*≈ -Δ*H* ^0^ *d*ξ, with ξ the extent of reaction.

2) The chemical potential is expressed as $\mu_{i}=\mu_{i}^{0}+R_{g}T\ln\left(f_{i}x_{i}\right)+z_{i}F\psi$ , where *R*_*g*_ is the gas constant, $\mu_{i}^{0}$ is the standard chemical potential, *f*_*i*_ the activity coefficient (accounting for nonideality), *z*_*i*_ the charge of component *i*, *x*_*i*_ the molar fraction, *F* the faraday constant and ψ the electric potential.

3) The variation of surface tension depends on local changes in temperature and concentration: $d\gamma =\gamma_{T}dT+\underset{i}{\sum }\gamma_{C_{i}}dC_{i}$ .

Taking the surface differential, multiplying by the area A and integrating along the surface ([Disp-formula eq8]), we obtain

9 $$\begin{array}{ll} E_{s}^{\left(e\right)}= & -A\Delta H^{0}\left\langle \nabla_{s}\xi \right\rangle +A\underset{i}{\sum }\left(\mu_{i}^{0}+R_{g}\left\langle T\ln\left(f_{i}x_{i}\right)\right)\nabla_{s}C_{i}\right\rangle \\ & +AF\underset{i}{\sum }z_{i}\left\langle \psi \nabla_{s}C_{i}\right\rangle -A\gamma_{T}\left\langle \nabla_{s}T\right\rangle -A\underset{i}{\sum }\gamma_{C_{i}}\left\langle \nabla_{s}C_{i}\right\rangle \end{array}$$

in which we have defined ∫_*S’*_ ... *dS’* = ⟨···⟩. As a consequence, only active particles that create asymmetric distributions of temperature or concentrations, due to an asymmetric reaction rate, develop excess energy, leading to active diffusion.

Furthermore, the dependence on surface gradients implies a coupling with reaction-induced surface mass and heat fluxes. This leads to the interpretation of surface energy excess, and consequently, active particle diffusivity, as being driven by the nondissipative currents within the overall dissipative process.^29,30^

#### Dissipative Approach

From the previous observation, we wonder whether the total entropy change might also encode information about a dissipative component of the surface energy. In this subsection, we explore this possibility by examining how entropy production at the interfaces contributes to energy conversion and transport, aiming to characterize a thermodynamically consistent framework for surface-driven activity.

To do this, we write the differential change of the excess entropy at the surface

10 $$\delta S=-R\underset{i}{\sum }\ln x_{i}dN_{i}+\delta \Sigma$$

in which δΣ is the irreversible change of the entropy at the surface. Taking the time derivative of ([Disp-formula eq10]) and defining the entropic change of the surface times the temperature as the dissipative surface energy $E_{s}^{\left(d\right)}$ , we have

11 $$\frac{E_{s}^{\left(d\right)}}{\tau }=-R_{g}\underset{i}{\sum }\left\langle T{Ṙ}_{i}\ln x_{i}\right\rangle +\left\langle T\sigma \right\rangle$$

with *Ṙ*_*i*_ the local reaction rate, σ the local entropy production rate and τ the characteristic time of the process.

Surface dissipative energy links the entropic cost associated with maintaining concentration gradients that emerge due to a chemical reaction with the intrinsic production of entropy at the interface. It is noteworthy that the activity driven by the surface reaction is thermodynamically sustained not only by the contribution of chemical energy, but also by the excess entropy derived from mixing and energy dissipation, which continuously reorganize matter and energy at the interface.

Finally, given the dissipative surface excess energy ([Disp-formula eq11]), the active diffusivity based on the dissipative approach is

(12 $$D^{\left(d\right)}=D_{0}\left(1+\frac{\left|E_{s}^{\left(d\right)}\right|}{k_{B}T}\right)$$

This perspective offers an alternative approach to exploring dissipation’s role in transport properties’ nontrivial behavior. This leads to an interpretation of excess surface dissipative energy—and thus the active diffusivity—as arising solely from the irreversible components of the dissipative process.^31^

## Model for a Janus Particle

We consider as our studied system the interface between a catalytic Janus particle and the surrounding fluid (Figure 1). The system operates in a nonequilibrium steady state, under the assumption that local thermodynamic equilibrium (LTE) holds at the interface. Our model involves a first-order surface reaction characterized by a kinetic constant that is independent of temperature. This approximation is valid when the interfacial thermal conductivity is high, the reaction enthalpy is low, or the temperature variations along the interface are minimal, though not entirely absent. A similar assumption is made for all parameters, which are considered constant due to the small variations in temperature and concentration, and the typically dilute nature of the system.

This assumption is justified because the suspension is dilute and immersed in a large thermal bath, so the bath temperature remains effectively constant and the surrounding fluid acts as an equilibrium reservoir. Under these conditions, the particle-fluid interface remains in local equilibrium throughout the transient relaxation process, which ultimately converges to equilibrium once the chemical fuel is consumed. These conditions have been extensively validated through nonequilibrium molecular simulations,^32–34^ which demonstrate that LTE holds reliably under such circumstances.

The key surface quantities are obtained by solving the corresponding conservation laws. We define the catalytic zone as -π/2 ≤ ϕ ≤ π/2 and assume symmetry of the fields with respect to θ = π/2 . Under these assumptions, and given the Janus nature of the catalytic particle, the relevant variables depend primarily on the azimuthal angle ϕ. Accordingly, we express the balance equations derived in Supporting Information C in terms of this reduced description and in dimensionless quantities.

The dimensionless substrate concentration at the particle interface *Ĉ*_*M*_ is given by:

13 $$0=\frac{\partial^{2}{Ĉ}_{M}}{\partial \phi^{2}}-\alpha^{2}{Ĉ}_{M}\Theta \left(\phi_{0}-\phi \right)-\beta^{2}\left({Ĉ}_{M}-{Ĉ}_{M}^{\left(b\right)}\right)$$

where α^2^ = *k*_*r*_ *R* ^2^/*D*_*s*_ and β^2^ = *UR* ^2^/*D*_*s*_ are the dimensionless numbers quantifying the reaction-diffusion and adsorption-diffusion effects on the interface. For the dimensionless product concentration *Ĉ*_*N*_, we have

14 $$0=\frac{\partial^{2}{Ĉ}_{N}}{\partial \phi^{2}}+\alpha^{2}{Ĉ}_{M}\Theta \left(\phi_{0}-\phi \right)-\beta^{2}{Ĉ}_{N}$$

We have assumed that the product concentration at the interface is much larger than its concentration in the bulk and that its physicochemical and transport properties are very similar to those of the substrate. The dimensionless temperature at the interface *T̂* fulfills the equation

15 $$0=\frac{\partial^{2}\mathrm{T̂}}{\partial \phi^{2}}+\Theta \left(\phi_{0}-\phi \right)\lambda^{2}{Ĉ}_{M}-\omega^{2}\left(\mathrm{T̂}-{\mathrm{T̂}}^{\left(b\right)}\right)$$

in which for an exothermic reaction, $\lambda^{2}=\frac{R^{2}\left|\Delta H_{r}\right|k_{r}C_{0}}{kT_{0}}$ , and $\omega^{2}=\frac{U_{q}R}{k}$ . These dimensionless numbers represent the effects of heat generation-conduction and cooling-conduction, respectively.

When the substrate and/or product are charged species, we define the dimensionless electric potential *ψ̂* from the Poisson–Boltzmann equation

16 $$\frac{\partial^{2}\mathrm{\psi ̂}}{\partial \phi^{2}}=-\xi^{2}\underset{i}{\sum }z_{i}{Ĉ}_{i}$$

with $\xi^{2}=\frac{R^{2}C_{0}N_{A}q_{0}^{2}}{k_{B}T\varepsilon_{0}}$ , *ψ̂* = ψ*q*_0_/*k*_*B*_ *T*. We have assumed a homogeneous charge distribution in the surrounding fluid, consistent with the dilute limit for Janus particles, where interparticle interactions are negligible. Within this approximation, the ions in solution contribute only a constant offset to the potential, without significantly modifying its spatial structure. At low ion concentrations, ionic interactions are weak, and the solvent can be treated as a continuous medium with nearly uniform ion distribution, as described by Debye–Hückel theory. The definitions of the physicochemical and transport parameters used to define the dimensionless numbers, together with their corresponding values for the cases studied, are provided in Table 3.

## Analytic Expressions for the Surface Excess Energy

The analytic expressions for each contribution of the surface excess energy ([Disp-formula eq9]) can be obtained from the analytic solution for the dimensionless fields presented in Supporting Information D.

In Table 1, we present the solution for each contribution of the surface excess energy for α^2^ ≪ 1 and β^2^ ≪ 1 . The first term in ([Disp-formula eq9]), $E_{s,r}^{\left(e\right)}$ (see Table 1), represents the energy effect due to chemical reaction. The second term in ([Disp-formula eq9]), $E_{s,S}^{\left(e\right)}$ , corresponds to the entropic contribution arising from spatial variations in surface concentration[Fn fn1].The third term in ([Disp-formula eq9]), $E_{s,\psi }^{\left(e\right)}$ , represents the self-electrophoretic part. The fourth term in ([Disp-formula eq9]), $E_{s,\gamma_{C_{i}}}^{\left(e\right)}$ , accounts for the self-diffusiophoretic effect, while the last term, $E_{s,\gamma_{T}}^{\left(e\right)}$ , describes the self-thermophoretic contribution. The magnitude of the surface excess energy is the absolute value of the sum of the components: $\left|E_{s}^{\left(e\right)}\right|=\left|E_{s,r}^{\left(e\right)}+E_{s,S}^{\left(e\right)}+E_{s,\psi }^{\left(e\right)}+E_{s,\gamma_{C_{i}}}^{\left(e\right)}+E_{s,\gamma_{T}}^{\left(e\right)}\right|$ .

**Table 1:** Surface Energy Contributions

Surface energy source	Result
$E_{s,r}^{\left(e\right)}$	$-A\epsilon \left(\Delta H_{r}+\Delta \mu_{r}^{0}\right)\frac{1-\sqrt{{\left(\alpha /\beta \right)}^{2}+1}}{1+\sqrt{{\left(\alpha /\beta \right)}^{2}+1}}\left\langle C_{M}\right\rangle$
$E_{s,S}^{\left(e\right)}$	*AεR_g_ T*_0_ In (⟨*C̃*_*M*_⟩(1 – *w*_0_))(*α* ^2^ + *β* ^2^)⟨*C_M_* ⟩
$E_{s,\psi }^{\left(e\right)}$	$\epsilon FA\frac{k_{B}T}{q_{0}}\xi^{2}\left[\frac{\left\langle {Ĉ}_{M}\right\rangle z_{M}}{w_{0}\beta }-\frac{\alpha^{2}}{\alpha^{2}+\beta^{2}}z_{N}\right]\left\langle C_{M}\right\rangle$
$E_{s,\gamma_{C_{i}}}^{\left(e\right)}$	$A\left(\gamma_{C_{M}}\frac{1-\sqrt{{\left(\alpha /\beta \right)}^{2}+1}}{1+\sqrt{{\left(\alpha /\beta \right)}^{2}+1}}+\gamma_{C_{N}}\frac{\alpha^{2}}{\alpha^{2}+\beta^{2}}\right)\left\langle C_{M}\right\rangle$
$E_{s,\gamma_{T}}^{\left(e\right)}$	$A\gamma_{T}\frac{T_{0}}{C_{0}}w_{0}\frac{\lambda^{2}}{\omega^{2}}\left(\alpha^{2}+2\beta^{2}\right)\left\langle C_{M}\right\rangle$

Our study reveals that the surface excess energy is proportional to the average surface substrate concentration, $\left\langle C_{M}\right\rangle =\frac{\beta^{2}}{\alpha^{2}+\beta^{2}}$ , which is directly related to the average reaction rate, *ṙ* = ⟨*J*_*r*_⟩ = *k*_*r*_ ⟨*C*_*M*_⟩. Furthermore, when the electrostatic contribution dominates, the excess energy scales with the square of the average substrate concentration, i.e., $E_{s}^{\left(e\right)}\propto \langle C_{M}\rangle^{2}$ .

It is important to emphasize that the excess energy is strongly influenced by the ratio between reaction and absorption α/β, and between heat production and cooling rate λ/ω. When absorption dominates, i.e., β ≫ α, self-thermophoresis becomes the primary source of surface excess energy. In contrast, in a reaction-dominated regime, i.e., β ≪ α, self-diffusiophoresis, self-electrophoresis, and enthalpic-free energy variations play the dominant role. For micrometric particles, α^2^ and β^2^ approach to one, making entropic effects increasingly significant. Additionally, for highly exothermic reactions with poor heat dissipation, i.e., λ^2^ ∼ ω^2^, self-thermophoresis is amplified and may become the main contributor to surface excess energy.

In Figure 3, we demonstrate the dependence of surface excess energy on the mean substrate concentration, as previously obtained in Table 1, via numerical solution of the balance equations presented in Supporting Information C. When considering only variations in product and substrate concentrations, as well as temperature, the dependence is linear with ⟨*C*_*M*_⟩ (as shown in Table 1 for $E_{s,r}^{\left(e\right)}$ , $E_{s,\gamma_{C_{i}}}^{\left(e\right)}$ , and $E_{s,\gamma_{T}}^{\left(e\right)}$ ), see Figure 3(a). In contrast, when accounting for the effect of the electrostatic field, the dependence becomes quadratic with ⟨*C*_*M*_⟩, as presented in Table 1 for $E_{s,\psi }^{\left(e\right)}$ . In the case of the entropic effect, since the dimensionless concentration is far from zero, it is expected a linear behavior as observed in Figure 3(b). This difference reflects the distinct physical mechanisms underlying each contribution. Variations in surface tension, and thus in the surface excess energy, convert changes in interfacial composition and temperature into mechanical stresses and therefore scale linearly with the average interfacial concentration ⟨*C*_*M*_⟩. By contrast, the electrochemical contribution depends not only on concentration variations but also on the electric field generated by the ionic species themselves. This charge-field coupling amplifies the energetic response, so that changes in ⟨*C*_*M*_⟩, and hence in the reaction rate *ṙ*, lead to a quadratic dependence of the surface excess energy.

**Figure 3:**
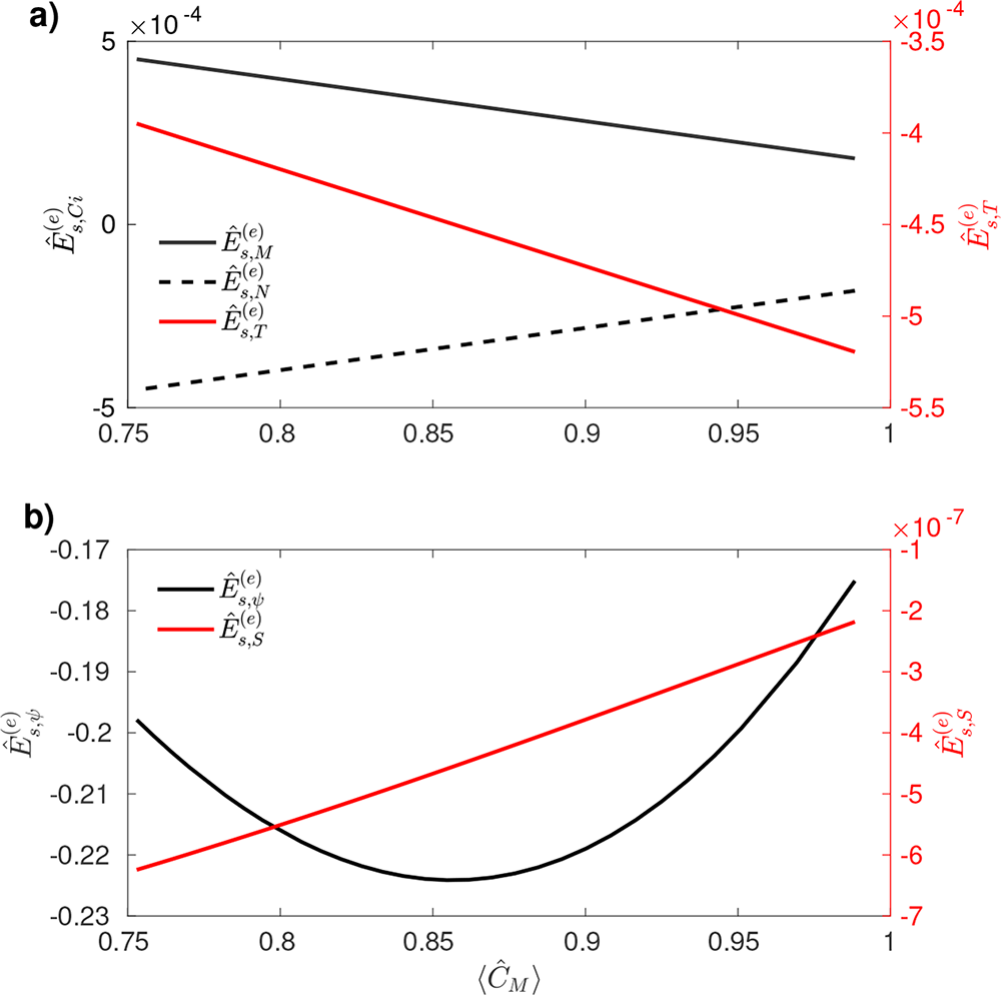
Dimensionless surface excess energy as a function of the mean substrate concentration. (a) Left *y*-axis: Contribution from variations in substrate and product concentrations: ${Ê}_{s,C_{i}}^{\left(e\right)}=\frac{1}{4\pi R^{2}}\int_{S}\nabla_{S}{Ĉ}_{i}dS$ . Right *y*-axis: Contribution from temperature variations: ${Ê}_{s,T}^{\left(e\right)}=\frac{1}{4\pi R^{2}}\int_{S}\nabla_{S}\mathrm{T̂}dS$ . (b) Left *y*-axis: Contribution from the electrostatic potential: ${Ê}_{s,\psi }^{\left(e\right)}=\frac{1}{4\pi R^{2}}\underset{i}{\sum }z_{i}\int_{S}\nabla_{S}\mathrm{\psi ̂}{Ĉ}_{i}dS$ . Right *y*-axis: Entropic contribution from variations in substrate and product concentrations ${Ê}_{s,S}^{\left(e\right)}=\frac{1}{4\pi R^{2}}\underset{i}{\sum }\int_{S}\ln{Ĉ}_{i}\nabla_{S}{Ĉ}_{i}dS$ .

In the context of the dissipative approach, Table 2 summarizes the contributions to the dissipative surface energy $E_{s}^{\left(d\right)}$ . Supporting Information E presents the entropy production rate and its individual contributions to $E_{s}^{\left(d\right)}$ . Specifically, $E_{s,S}^{\left(d\right)}$ corresponds to the entropic contribution from mixing, $E_{s,D}^{\left(d\right)}$ accounts for dissipation due to mass diffusion, $E_{s,Q}^{\left(d\right)}$ represents the contribution from heat conduction, $E_{s,r}^{\left(d\right)}$ is the dominant term associated with energy dissipation from the chemical reaction, and $E_{s,v}^{\left(d\right)}$ captures the dissipation due to surface velocity gradients. The table reveals both linear and quadratic dependencies on the average concentration. Among the terms, the entropy of mixing and the energy dissipated by the chemical reaction are expected to dominate, as both scale proportionally with α^2^.

**Table 2:** Dissipative Surface Energy Contributions

Effect	Result
$E_{s,S}^{\left(d\right)}$	–*Aετk*_*r*_ *R*_*g*_ *T*_0_ln(⟨*Ĉ*_*M⟩*_(1–*w*_0_))(α^2^+β^2^)⟨*C*_*M*_⟩
$E_{s,D}^{\left(d\right)}$	$A\varepsilon \frac{\tau D_{M}R_{g}T_{0}}{R^{2}}\beta^{2}\left(\frac{\pi^{2}}{24C_{0}}\beta^{2}\left\langle C_{M}\right\rangle +\frac{2}{3}\alpha^{4}\pi^{2}\right)\left\langle C_{M}\right\rangle$
$E_{s,Q}^{\left(d\right)}$	$A\varepsilon \frac{\tau D_{M}}{R^{2}}\alpha^{2}\beta^{2}\pi^{2}\frac{\lambda^{2}}{24\omega^{2}}\Delta H_{r}\left\langle C_{M}\right\rangle$
$E_{s,r}^{\left(d\right)}$	$A\varepsilon \frac{\tau D_{M}R_{g}T_{0}}{R^{2}C_{0}}\frac{{\sqrt{{\left(\alpha /\beta \right)}^{2}+1}}^{3}}{\left(1+{\sqrt{{\left(\alpha /\beta \right)}^{2}+1}}^{3}\right)}\alpha^{2}\langle C_{M}\rangle^{2}$
$E_{s,v}^{\left(d\right)}$	$\frac{\varepsilon \tau }{\eta_{s}}[\beta^{4}\gamma_{C_{M}}^{2}+\alpha^{4}\gamma_{C_{N)}}^{2}+\lambda^{4}\frac{T_{0}^{2}}{C_{0}^{2}}\gamma_{T}^{2}+({\frac{Fk_{B}TR}{q_{0}}}^{2})\left(z_{M}^{2}+2\alpha^{2}z_{M}z_{N}+\alpha^{4}z_{N}^{2}\right)]\langle C_{M}\rangle^{2}$

## Results and Discussion

To validate our theoretical framework, we analyze two experimentally studied systems where active diffusivity has been observed to depend on the reaction rate. The first case considers nanometric catalytic Janus particles that utilize a charged salt as a substrate,^20^ while the second examines phospholipid vesicles with embedded enzymes hydrolyzing ATP.^25^ To obtain the results presented in Figures 4 and 5, we used the experimental parameter values (*R*, *T*_0_, *k*_*r*_, *z*_*M*_, *z*_*N*_, Δ*H*_*r*_, Δμ_*r*_) provided in refs. 20,25 The dependencies of surface tension on concentration and temperature ( $\gamma_{C_{M}}$ , $\gamma_{C_{N}}$ , γ_*T*_), as well as diffusivities, were extracted from the literature.^35,36,40,41^ Thermal conductivity, electric permittivity, and interface thickness, were estimated using data of refs. 35,36,40,41 Finally, to vary the average concentration and, consequently, the average reaction rate, as reported in experiments, we varied the substrate bulk concentration. The symbols and numerical values of the parameters used to obtain the model results are summarized in Table 3. The key fitting parameter in our model is the interfacial thickness *ε*. In addition, three other relevant fitting parameters—the derivatives of the surface tension with respect to concentration and temperature ( $\gamma_{C_{M}}$ , $\gamma_{C_{N}}$ , γ_*T*_)—are varied within 10% to 50% of the values reported in the literature for bulk systems, while preserving their order of magnitude. It is worth noting that these quantities are intrinsically difficult to define, as they should account for interactions with the particle surface. However, since neither substrates nor products adsorb onto the particle, we assume an ideal behavior close to bulk conditions.

**Table 3:** Parameter Definition and Values for Study Case 1 (Nanometric Catalytic Janus Particles) and Case 2 (Phospholipid Vesicles with Embedded Enzymes)

Symbol	Parameter	value case 1 ^20^	value case 2 ^25^
*C*_0_	Bulk concentration	0.5 mM	0.5 mM
*T*_0_	Bulk temperature	298.15 K	294.15 K
*k*_*r*_	Kinetic constant	21.4 s^–1^	250 s^–1^
*D*_*s*_	Interface diffusivity	650 μm^2^/s^35^	360 μm^2^/s^36^
η_*s*_	Interface viscosity	0.891 mPa s^–1^	1 mPa s^–1^
*U*	Mass transfer coefficient	10 s−1 ^37^	10 s−1 ^37^
Δ*H*_*r*_	Reaction heat	–91 kJ/mol	–30 kJ/mol^38^
$\Delta \mu_{r}^{0}$	Reaction free energy	30 kJ/mol^39^	–60 kJ/mol^38^
κ	Thermal conductivity	0.6 W/Km	0.59 W/Km
*U*_*q*_	Heat transfer coefficient	0.01 W/Km2 ^37^	0.01 W/Km2 ^37^
ϵ^a^	Interface thickness	5 nm	15 nm
*z*_*M*_	Substrate charge	–3	0
*z*_*N*_	Product charge	–4	–1
$\gamma_{C_{M}}$ ^b^	γ derivative with *C*_*M*_	1.5 mJ/m^2^M^40^	–0.01 mJ/m^2^M^41^
$\gamma_{C_{N}}$ ^b^	γ derivative with *C*_*N*_	0.2 mJ/m^2^M^40^	0.02 mJ/m^2^M^41^
γ_*T*_ ^b^	γ derivative with *T*	–0.2 mJ/m^2^K^40^	–0.01 mJ/m^2^K^41^
*D*_0_	Passive particle diffusivity	7.75 μm^2^/s	0.065 μm^2^/s
*R*	Particle radius	9 nm	2.1 μm

a Main fitting parameter. Estimated values.

b Secondary fitting parameters. Modified values from literature.

**Figure 4:**
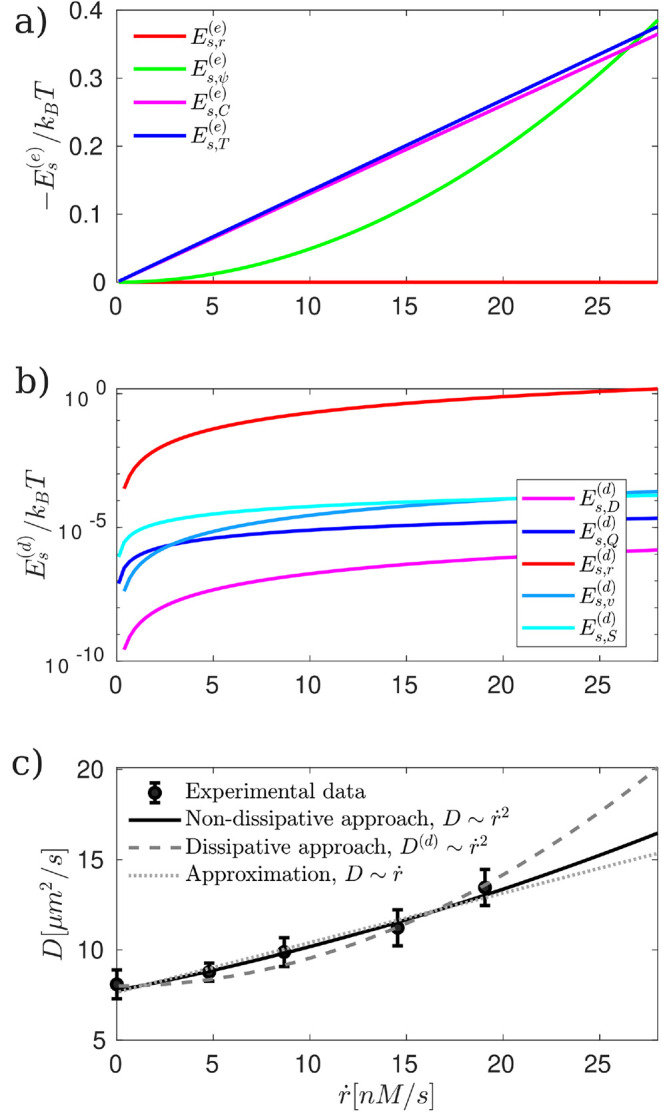
Nondissipative and dissipative surface excess energy and active diffusivity of nanometric Janus particles as a function of the average reaction rate *ṙ* = *k*_*r*_⟨*C*_*M*_⟩[nM/s]. (a) Negative surface excess energy -*E*_*s*_^*(e)*^/*k*_*B*_ *T* computed from eq ([Disp-formula eq9]). (b) Dissipative surface excess energy $E_{s}^{\left(d\right)}/k_{B}T$ computed from eq ([Disp-formula eq11]). (c) Active diffusivity *D* computed from eq ([Disp-formula eq6]) (continuous black line), active diffusivity computed from dissipative approach *D* ^*(d)*^ from eq ([Disp-formula eq12]) (dashed dark gray line) whereas the black dots with the error bars represent the experimental data,^20^ all following a quadratic dependence with *ṙ*. The dotted light gray line corresponds to the approximation proposed in ref.^20^ which considers only the self-thermophoretic effect, and follows a linear dependence on *ṙ*. The model results are shown for 1 × 10^–19^ ≤ λ^2^ ≤ 1.13 × 10^–17^, 3 × 10^–4^ ≤ ξ^2^ ≤ 3.29 × 10^–2^ and τ = (*k*_*r*_β^2^)^−1^.

**Figure 5:**
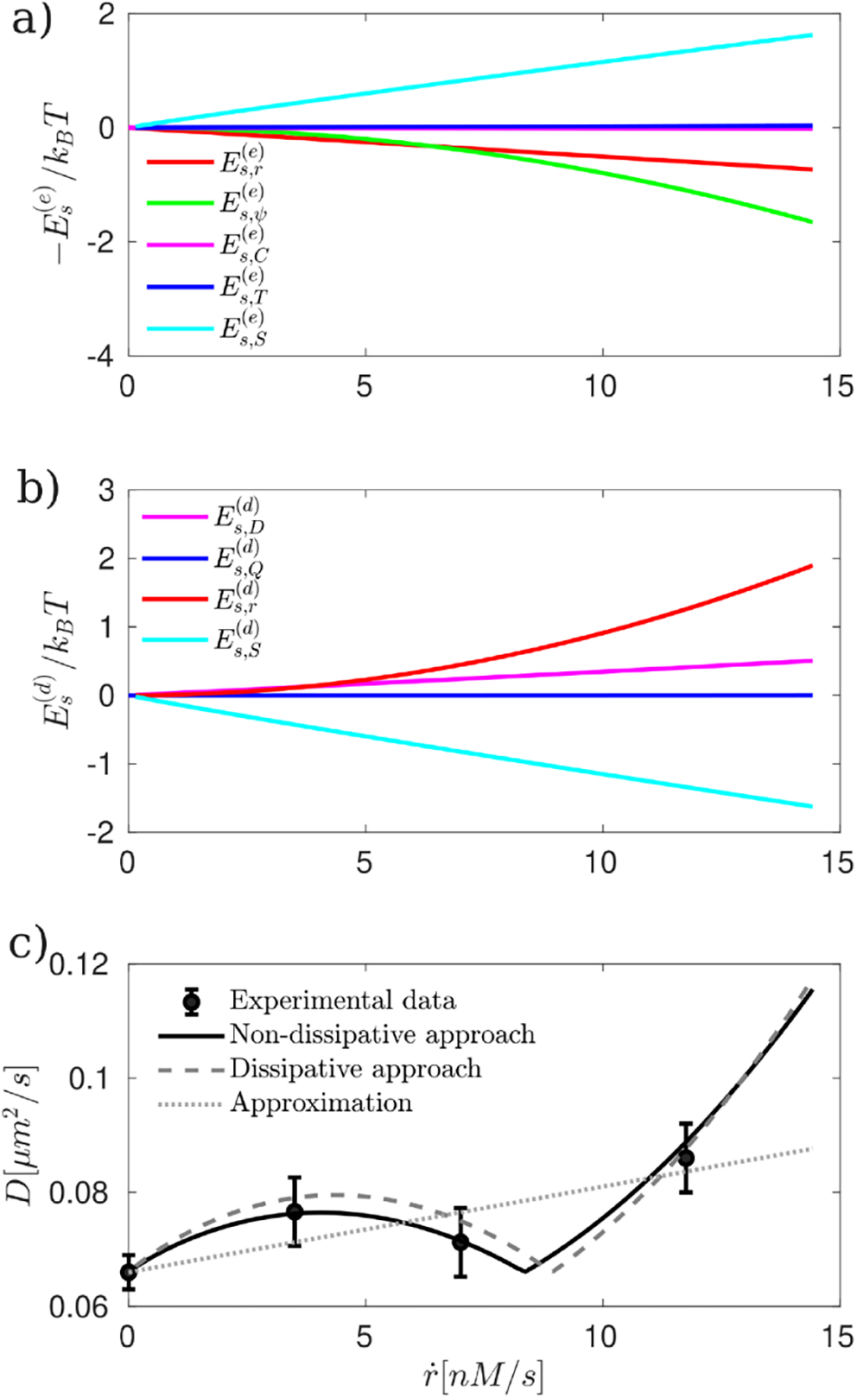
Nondissipative and dissipative surface excess energy and active diffusivity of catalytic liposomes as a function of the average reaction rate *ṙ* = *k*_*r*_⟨*C*_*M*_⟩[nM/s]. (a) Negative surface excess energy $-E_{s}^{\left(e\right)}$ ­[J] computed from eq ([Disp-formula eq9]). (b) Dissipative surface excess energy $E_{s}^{\left(d\right)}/k_{B}T$ computed from eq ([Disp-formula eq11]).(c) Active diffusivity *D* computed from eq ([Disp-formula eq6]) (continuous black line), active diffusivity computed from the dissipative approach *D* ^*(d)*^ from eq ([Disp-formula eq12]) (dashed dark gray line), whereas the black dots with error bars correspond to experimental data from ref.^25^ The dotted light gray line corresponds to a linear approximation without considering entropic effects. For 1 × 10^–15^ ≤ λ^2^ ≤ 2 × 10^–13^,3 × 10^–2^ ≤ ξ^2^ ≤ 2.9, and $\tau =k_{r}^{-1}$ .

We demonstrate that the increase in active diffusivity is a direct consequence of the rise in surface excess energy with the reaction rate. Crucially, we identify the average surface substrate concentration as the key parameter governing this effect. Furthermore, our analysis shows that surface excess energy is influenced not only by the reaction rate but also by the reaction heat, affinity, particle size, surface tension variations, and the charge of chemical compounds at the interface. This comprehensive understanding allows us to bridge experimental observations with a theoretical foundation, offering new insights into the underlying mechanisms driving enhanced mobility in active systems.

### Nanometric Catalytic Janus Particles

In Figure 4, we present the surface excess energy and active diffusivity of nanometric Janus particles. Figure 4(a) illustrates the behavior and relative contributions of the different terms in the surface excess energy. The blue line represents the self-thermophoretic contribution $E_{s,T}^{\left(e\right)}$ , the magenta line corresponds to the self-diffusiophoretic term $E_{s,\gamma_{C}}^{\left(e\right)}$ , the green line depicts the self-electrophoretic term $E_{s,\psi }^{\left(e\right)}$ , and the red line represents the reaction-phoretic contribution $E_{s,r}^{\left(e\right)}$ . We observe that both self-thermophoretic and self-diffusiophoretic terms dominate, exhibiting similar trends and magnitudes. Consequently, the enhancement of active diffusivity (Figure 4(c)) is not solely due to self-thermophoretic effects, as previously suggested in ref.^20^ but also significantly influenced by self-diffusiophoretic contributions.

In Figure 4(b), we present the contributions to the dissipative surface excess energy on a logarithmic scale. It is evident that the energy dissipation associated with the chemical reaction, $E_{s,r}^{\left(d\right)}$ (red line)-a scalar process-dominates at the nanoscale. In contrast, the contributions from vectorial and tensorial processes, such as mass diffusion $E_{s,D}^{\left(d\right)}$ (magenta line), heat conduction $E_{s,Q}^{\left(d\right)}$ (blue line), and fluid flow $E_{s,v}^{\left(d\right)}$ , are negligible. Additionally, the entropic contribution from mixing, $E_{s,S}^{\left(d\right)}$ , is also minor compared to the entropy produced by irreversible processes. These results indicate that, from a dissipative perspective, the enhancement of diffusivity observed in Figure 4(c) is primarily driven by the energy dissipation associated with the chemical reaction occurring at the nanoparticle surface.

In Figure 4(c), the continuous black lines and dashed dark gray line shows our theoretical predictions from the nondissipative and dissipative approaches, respectively, following a quadratic dependence on the reaction rate whereas the light gray dotted line shows a linear approximation,^20^ when considering γ_*T*_ = −0.5J/m^2^K, 3 orders of magnitude larger than literature estimates. The black dots with error bars correspond to experimental data from ref.^20^ which follows a quadratic behavior (with *R*_*r*_ = 0.993). We observe that self-electrophoresis plays a crucial role, scaling quadratically with the average reaction rate and improving agreement between model results and experimental data. Notably, self-thermophoresis alone cannot fully explain the experimental results, as it predicts a linear dependence of *D* on *ṙ* and requires a γ_*T*_ value far from physical meaning. Regarding the dissipative approach, it is worth noting that it captures the quadratic dependence on the reaction rate and provides a sufficiently accurate fit to the experimental data when considering the characteristic time τ = (*k*_*r*_ *β* ^2^)^−1^. This implies that, at the nanoscale, the relevant timescales must account for the interplay between reaction kinetics and diffusion along the interface. The key insight is that the energy dissipated during the chemical reaction sustains the gradients required to increase the surface energy, thereby enhancing the particle’s diffusivity.

The observed discrepancy between the dissipative and nondissipative approaches, particularly at high reaction rates, indicates that computing the dissipative surface excess energy, for nanometric particles, solely from entropy changes—both reversible and irreversible—may not fully capture the system’s behavior. This suggests that entropy production and mixture entropy alone are insufficient to describe the dynamics at large activities for such small systems. Under these conditions, additional non-dissipative contributions, such as those captured by the concept of frenesy, become important.^30^ A comprehensive description should therefore combine both dissipative contributions (entropy changes) and nondissipative effects (frenesy), providing a unified framework capable of explaining the system’s behavior across the full range of activities.

### Phospholipid Vesicles with Embedded Enzymes

In Figure 5, we present the surface excess energy and active diffusivity of phospholipid vesicles with embedded enzymes. Figure 5(a) illustrates the behavior and relative contributions of the different terms in the surface excess energy. The blue line represents the self-thermophoretic contribution $E_{s,T}^{\left(e\right)}$ , the magenta line corresponds to the self-diffusiophoretic term $E_{s,\gamma_{C}}^{\left(e\right)}$ , the green line depicts the self-electrophoretic term $E_{s,\psi }^{\left(e\right)}$ , the red line represents the reaction-phoretic contribution $E_{s,r}^{\left(e\right)}$ , and the cian line corresponds to the entropic contribution $E_{s,S}^{\left(e\right)}$ . Unlike the previous case of the nanometric catalytic Janus, entropic contributions become significant due to the considerable increase in particle size, increasing α^2^ and β^2^. On the other hand, both self-thermophoretic and self-diffusiophoretic contributions are negligible, as the surface tension exhibits weak dependence on substrate and product concentrations as well as temperature. Consequently, entropic, enthalpic, free energy, and self-electrophoretic effects dominate and compete among them, leading to a nonmonotonic behavior of active diffusivity *D* as a function of the mean reaction rate (Figure 5(c)). This nonmonotonicity arises because the self-electrophoretic and reaction-phoretic contributions can become negative depending on parameters such as the charge (*z*_*M*_ and *z*_*N*_) and concentration of reactants and products (*C*_*M*_ and *C*_*N*_), the signs of the derivatives of surface tension with respect to temperature (γ_*T*_) and concentration ( $\gamma_{C_{M}}$ and $\gamma_{C_{N}}$ ), and the sign of the reaction’s free energy change (Δμ_*r*_). Variations in these parameters modulate the relative magnitude and sign of each contribution, producing the observed complex dependence of *D* on the mean reaction rate. Note that the local maximum corresponds to the regime in which the self-electrophoretic contribution becomes significant, whereas the minimum occurs when the entropic contribution cancels the self-electrophoretic and reaction-phoretic contributions.

In Figure 5(b), we show the contributions to the dissipative surface excess energy. In this case, the entropic contribution from mixing, $E_{s,S}^{\left(d\right)}$ (cyan line), and the dissipative contributions from the chemical reaction, $E_{s,r}^{\left(d\right)}$ (red line), and mass diffusion, $E_{s,D}^{\left(d\right)}$ (magenta line), are found to be of comparable magnitude. This results in a competition among these effects, giving rise to the nonmonotonic behavior of *D* ^*(d)*^ observed in Figure 5(c). Both the dissipative and nondissipative approaches lead to the same active diffusion behavior.

In Figure 5(c) the continuous black line and dashed dark gray line show our theoretical predictions, whereas the dotted light gray line shows a linear approximation when considering $\gamma_{C_{M}}=-1\mathrm{mJ}/m^{2}K$ or $\gamma_{C_{M}}=1\mathrm{mJ}/m^{2}K$ and neglecting entropic effects. The black dots with error bars correspond to experimental data from ref.^25^ Notably, around *ṙ* = 8.1 nM/s, the active diffusivity reaches its minimum value, corresponding to the point where the surface excess energy cancels. The small shift observed in Figure 5(c) between the nondissipative and dissipative approaches arises from the value of the dimensionless time $\tau =k_{r}^{-1}$ , which may differ slightly from the experimental value.

We observe that the enhancement of active diffusivity (Figure 5(c)) is not driven by self-diffusiophoretic effects, as previously suggested in ref.^25^ but rather by self-electrophoretic and reaction-phoretic contributions, or, from the energy dissipated by the chemical reaction. Moreover, self-electrophoresis remains significant, as it scales quadratically with the average reaction rate, leading to improved agreement with experimental results, (see Figure 5(c)). The entropy contribution in Figure 4(a) and the fluid flow contribution in Figure 5(b) are not shown because they are negligible compared to the other contributions; we exclude them to keep the figures uncluttered.

## Conclusions

We have shown that the active diffusivity of catalytic nanoparticles and enzyme-functionalized vesicles is governed by the excess surface energy generated by chemical reactions. This quantity was evaluated using two complementary perspectives: a nondissipative approach, based on differences between internal and surface energy, and a dissipative approach, based on the energy irreversibly lost at the interface.

For nanometric Janus particles, self-thermophoresis and self-diffusiophoresis contribute similarly to the active diffusion, while self-electrophoresis introduces a quadratic dependence on reaction rate that matches experimental trends. The dissipative analysis reproduces this quadratic behavior through reaction-driven energy dissipation at the surface.

For enzyme-functionalized vesicles, competing effects—mixing entropy, electrostatics, and reaction heat—give rise to a nonmonotonic dependence of the active diffusivity on the reaction rate, in a regime where self-diffusiophoretic and self-thermophoretic contributions are negligible. Within the dissipative analysis, this nonmonotonic behavior is explained by the competition between mixing entropy and entropy production. This behavior may be of interest for future applications, as the local maximum in the active diffusivity occurs when the self-electrophoretic (or entropy production in the dissipative perspective) contribution becomes significant. More importantly, the reaction rate that optimizes the diffusivity can be determined analytically using the expressions reported in Tables 1 and 2.

Overall, both approaches converge to consistent predictions and highlight excess surface energy as the central quantity controlling active diffusion. Multiple phoretic and entropic mechanisms act collectively, and their coupling must be considered rather than treated independently. Enhanced mobility ultimately arises from surface-tension variations sustained by entropy production, identifying excess surface energy as a key design principle for synthetic microswimmers and active colloids.

## Supplementary Material



## Footnotes

1 For. $w_{0}={\left(1+\sqrt{{\left(\alpha /\beta \right)}^{2}+1}\right)}^{-1}$ .

## Notes

Theoretical and analytical foundations underlying the main results of the manuscript; interfacial balance equations for mass, heat, and electrostatic potential, together with analytical solutions for sub strate, product, temperature, and electric fields on the particle surface; entropy production at the interface within a nonequilibrium thermodynamic framework, identifying the relevant flux–force pairs governing active diffusion­(PDF)
