# Supplementary material for: Surface Excess Energy as a Unifying Thermodynamic Framework for Active Diffusion
Source: J Phys Chem B. 2026 Jan 23;130(5):1694–703. doi: 10.1021/acs.jpcb.5c07096 (PMC12884529; doi:10.1021/acs.jpcb.5c07096)
Supplement: Supplementary file 1 [file jp5c07096_si_001.pdf]

Supporting Information: Surface Excess Energy as a Unifying Thermodynamic Framework for Active Diffusion

Andrés Arango-Restrepo*,† and J. Miguel Rubi†,‡

†*Departament de Física de la Matèria Condensada, Facultad de Fisica, Universitat de Barcelona, Barcelona, 08028, Spain*

‡*Institut de Nanociencia i Nanotecnologia, Universitat de Barcelona, 08028, Barcelona, Spain*

E-mail: aarangor@unal.edu.co

Abstract

This Supplementary Information provides the theoretical and analytical foundations underlying the main results of the manuscript. We first derive the orientation correlation function of an active particle in the overdamped rotational limit, clarifying the role of stochastic torques and the absence of phoretic alignment at long times. We then compute the mean translational kinetic energy from the Langevin dynamics, establishing the connection between phoretic forces and surface excess energy. Subsequent sections present the interfacial balance equations for mass, heat, and electrostatic potential, together with analytical solutions for substrate, product, temperature, and electric fields on the particle surface. These results are used to explicitly compute the surface excess energy and its dependence on reaction rate. Finally, we derive the entropy production at the interface within a nonequilibrium thermodynamic framework, identifying the relevant flux–force pairs governing active diffusion.

A: Orientation Correlation Function in the Overdamped Limit

We consider a spherical particle whose orientation is described by the unit vector $\mathbf{n}(t)$, evolving under stochastic rotational dynamics. Starting from the underdamped Langevin equation for the angular velocity (Eq.(2)), with $\mathbf{T}_{ph} = 0$, the overdamped limit is obtained by neglecting the inertial term $I\dot{\boldsymbol{\omega}}$. In this limit, the angular velocity becomes a zero-mean Gaussian process with correlation $\langle \omega_i(t)\omega_j(t')\rangle = 2D_r\delta_{ij}\delta(t-t')$, with D_r the rotational diffusion coefficient. The orientation vector then evolves according to Eq. (3). This formulation clarifies that the delta-correlation of $\boldsymbol{\omega}$ arises directly from the white-noise character of the random torque $\mathbf{T}_r$ through the overdamped Langevin dynamics.

Over time, the particle orientation vector undergoes random rotations due to stochastic torques. The dynamics correspond to a rotational diffusion process, for which the orientation correlation decays exponentially:

$$\langle \mathbf{n}(t) \cdot \mathbf{n}(t')\rangle = e^{-2D_r|t-t'|}. \tag{S.1}$$

This is valid in the limit where inertial effects are negligible and the particle's orientation undergoes a Markovian random walk on the particle.[1]

Since the system is isotropic (no preferred direction), we can compute the tensorial two-time correlation function:[2,3]

$$\langle n_i(t)n_j(t')\rangle = \left[\frac{1}{3} + \left(\langle \mathbf{n}(t) \cdot \mathbf{n}(t')\rangle - \frac{1}{3}\right)\right]\delta_{ij}. \tag{S.2}$$

Regarding the phoretic torque, in the absence of a preferred direction the self-induced local fields either align with a unit director vector, $\mathbf{m} \to \mathbf{n}$ (when the rotational relaxation time is sufficiently short for a gradient to develop), or remain completely random, $\mathbf{m}$, such that $\psi \to 0$. In both cases, the phoretic torque vanishes, either $\mathbf{T}_{ph} \to 0$ at all times, or $\langle \mathbf{T}_{ph}\rangle \to 0$ over long times. Consequently, $|\mathbf{T}_{ph}| \ll |\mathbf{T}r|$ at long times, and the phoretic torque becomes negligible compared to the random torque.

B: Mean translational kinetic energy

The solution of the Langevin equation (Eq.(1)) in 1-D is

$$v(t) = e^{-\xi_t t/m} v(0) + \int_0^t dt' e^{-\xi_t (t-t')/m} (F_{ph}(t') + F_t(t')) \tag{S.3}$$

Multiplying this expression by $v(t)$ and taking the ensemble average, we obtain:

$$\begin{aligned} \langle v(t)^2 \rangle =& e^{-2\xi_t t/m} v(0)^2 + \\ & \frac{1}{m^2} \int_0^t dt' e^{-\xi_t (t-t')/m} \int_0^t dt'' e^{-\xi_t (t-t'')/m} H \end{aligned} \tag{S.4}$$

in which $H = \langle (F_{ph}(t') + F_t(t'))(F_{ph}(t'') + F_t(t'')) \rangle$ and have used the fact that $\langle F_{ph}(t') + F_t(t') \rangle = \langle F_{ph}(t'') \rangle + \langle F_t(t'') \rangle = 0$, since the noise sources are not correlated because stochasticity of the phoretic force originates from the orientation vector $\mathbf{n}$, governed by Eq. (3), with a rotational noise source $\mathbf{T}_r$ independent of the translational noise source $\mathbf{F}_t$. Using this fact again, we have

$$\begin{aligned} H &= \langle F_{ph}(t') F_{ph}(t'') \rangle + \langle F_t(t') F_t(t'') \rangle \\ &= 2\xi_t B + 2\xi_t k_B T \end{aligned} \tag{S.5}$$

Substituting this expression in Eq.(S.4), and solving the integral, we obtain:

$$\langle v(t)^2 \rangle = e^{-2\xi_t t/m} v(0)^2 + 2\frac{B + k_B T}{m} \left(1 - e^{-2\xi_t t/m}\right) \tag{S.6}$$

The mean squared velocity at equilibrium ($t \to \infty$) is then the sum of the phoretic and thermal contributions

$$\langle v(t)^2 \rangle_{t\to\infty} = 2\frac{B}{m} + 2\frac{k_B T}{m} \tag{S.7}$$

The mean translational kinetic energy of the particle is then

$$\frac{m}{2} \langle v(t)^2 \rangle_{t\to\infty} = B + k_B T \tag{S.8}$$

and by considering that the surface excess energy $\left|E_s^{(e)}\right|$ is the source of energy of the phoretic force, we find that $B = \left|E_s^{(e)}\right|$.

C: Balance equations

The substrate concentration at the interphase between an AP and the bulk fulfills the substrate mass conservation equation

$$\mathbf{v}_s \cdot \nabla_s C_M = -\nabla_s \cdot \mathbf{J}_M - k_r C_M \Theta(\Omega_0 - \Omega) - U(C_M - C_M^{(b)}) \tag{S.9}$$

On the left-hand side, the term represents the advective contribution to substrate transport along the interface. On the right-hand side, the first term captures the diffusive contribution driven by concentration, temperature, and electric field gradients; the second term accounts for the chemical reaction contribution; and the final term describes mass transfer in the radial direction. Here $\mathbf{v}_s$ is the surface velocity of the interface, k_r is the reaction constant, U stands for the mass transfer coefficient, $C_M^{(b)}$ is the bulk substrate concentration and Ω_0 the borderline between both sides of the particle. The diffusive current is given by[4]

$$\mathbf{J}_M = -D_M \nabla_s C_M - D_M \mathscr{S}_s C_M \nabla_M T - D_M \frac{q_0 z_M C_M}{k_B T} \nabla_s \psi \tag{S.10}$$

in which D_M denotes the diffusivity of the substrate at the interface, $\mathscr{S}_M$ the substrate Soret coefficient and q_0 the electron charge per mole. The product concentration balance equation is

$$\mathbf{v}_s \cdot \nabla_s C_N = -\nabla_s \cdot \mathbf{J}_N + k_r C_M \Theta(\Omega_0 - \Omega) - U(C_N - C_N^{(b)}) \tag{S.11}$$

with $\mathbf{J}_N$ similar to $\mathbf{J}_M$ considering the transport properties of the product instead of the substrate. Since the reaction kinetics depends only on the substrate concentration, the energy balance at the interface is:

$$\mathbf{v}_s \cdot \nabla_s T = \kappa \nabla_s^2 T - k_r C_M \Delta H_r \Theta(\Omega_0 - \Omega) - U_q(T - T^{(b)}) \tag{S.12}$$

The left-hand side term corresponds to the advective contribution to the heat transport along the interface. On the right-hand side, the first term accounts for the diffusive contribution, the second represents the heat produced (or consumed) due to the exothermic (endothermic) chemical reaction, and the last term corresponds to the heat transfer in the radial direction. κ denotes the heat diffusivity constant at the interface, ΔH_r is the reaction enthalpy (negative for exothermic, and positive for endothermic reactions), U_q stands for the heat transfer coefficient, and $T^{(b)}$ is the bulk temperature. Note that we explicitly include tangential and normal transport contributions, with mass adsorption/desorption (last terms in Eqs.(S.9) and (S.11)) and heat flow (Eq.(S.12)) occurring in the direction normal to the interface.

The electrostatic potential ψ at a particle–fluid interface can be obtained from the Poisson equation

$$\nabla_s^2 \psi = -\frac{q_0}{\varepsilon} \sum_i z_i C_i \tag{S.13}$$

with ε the permittivity of the medium.

D: Analytical expressions for the fields

Our goal is to explicitly demonstrate the dependence of active diffusivity (and surface excess energy) on the mean substrate concentration and, consequently, on the mean reaction rate. We will obtain an analytical solution to the balances in Appendix C. Dimensional analysis shows that the thermodiffusion and electrodiffusion of the substrate and product on the surface (Eq. S.10) can be neglected when the diffusivities of both species are similar, their charges are exactly opposite, and the Soret coefficient is lower than 1.

In which $w_1 = \frac{\beta^2\left(\left(1-\sqrt{(\alpha/\beta)^2+1}\right)^{-2}-1\right)-\alpha^2}{\beta^2(\alpha^2+\beta^2)}$.

To confirm the assumption of symmetric fields around $\theta = \pi/2$, we numerically solve the surface balance equations using finite-difference methods implemented in MATLAB. Additionally, we analyze how the contributions to surface excess energy depend on the mean substrate concentration. To ensure the generality of our results, we analyzed the variations in the dimensionless quantities.

Table S1: Analytic solutions of the balance equations.

Variable	Catalytic side	Non-catalytic side
C_M/C_0	$\frac{\beta^2}{\alpha^2+\beta^2}+2k^+\cosh\left(\sqrt{\alpha^2+\beta^2}\phi\right)$	$2k^-\exp(-\beta\pi)\cosh(\beta(\pi-\phi))$
C_N/C_0	$4\frac{\alpha^2k^+}{\alpha^2+\beta^2}\cosh\left(\sqrt{\alpha^2+\beta^2}\phi\right)+m^+\cosh(\beta\phi)$	$2m^-\exp\{(-\beta\pi)\}\cosh(\beta(\pi-\phi))$
T/T_0	$\frac{k^+\lambda^2}{\omega^2}\cosh\left(\sqrt{\alpha^2+\beta^2}\phi\right)+n^+\cosh(\omega\phi)$ $+\frac{\lambda^2\beta^2}{\omega^2(\alpha^2+2\beta^2)}(1+2\alpha^2)+1$	$2n^-\exp\{(-\omega\pi)\}\cosh(\omega(\pi-\phi))+1$
$-\psi/\psi_0\xi^2$	$\frac{\beta^2}{\alpha^2+\beta^2}(\phi^2-\pi\phi-w_1)-\frac{2k^+}{\alpha^2+\beta^2}\cosh\left(\sqrt{\alpha^2+\beta^2}\phi\right)$	$2\frac{k^-}{\beta^2}\exp\{(-\beta\pi)\}\cosh(\beta(\pi-\phi))$

In Fig. S1(a), we observe that the substrate concentration is higher on the non-catalytic side (back

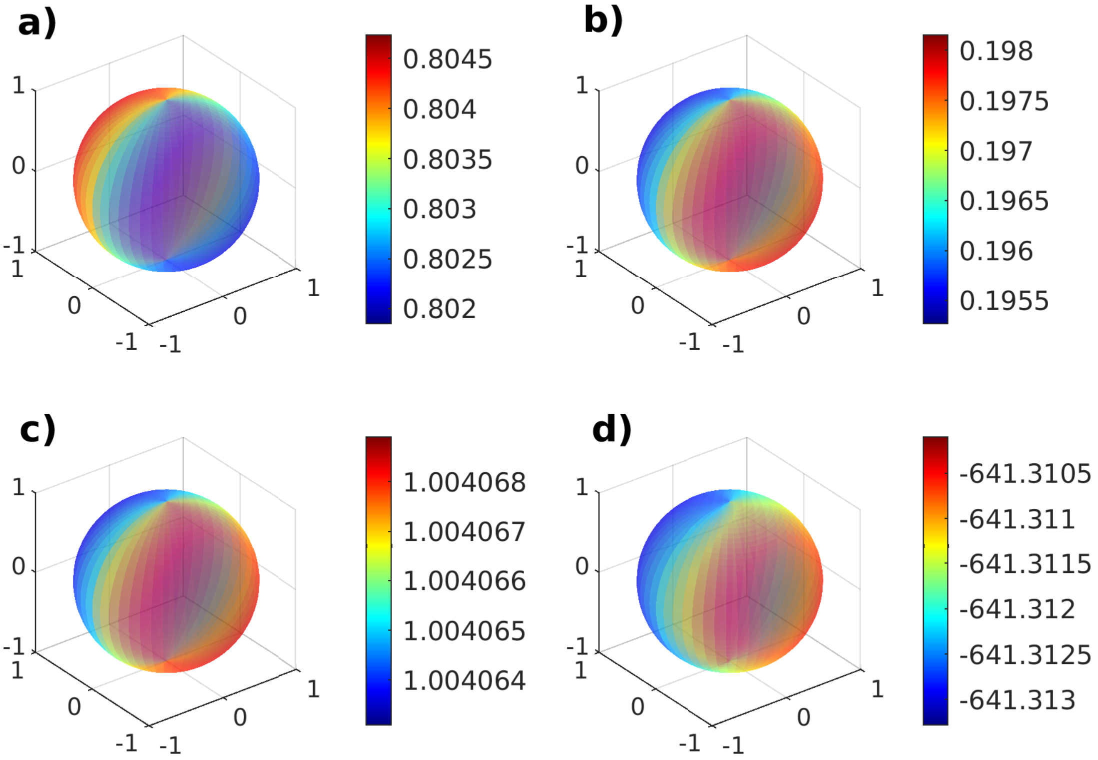

Figure S1: Surface fields. a) Dimensionless substrate concentration $\hat{C}_M = C_M/C_0$, b) Dimensionless product concentration $\hat{C}_N = C_N/C_0$, c) Dimensionless temperature $\hat{T} = T/T_0$, d) Dimensionless electrostatic potential $\hat{\psi} = \psi q_0/k_BT$. These results are obtained for $\alpha^2 = 10^{-2}$, $\beta^2 = 10^{-3}$, $\lambda^2 = 10^{-7}$, $\omega^2 = 10^{-5}$. Particle dimensions are also dimensionless, $\hat{r} = r/R$.

of the sphere) compared to the catalytic side (front of the sphere), as expected. Conversely, in

Fig. S1(b) and (c), the highest product concentration and temperature are reached on the front side, where the reaction occurs, generating both product and heat. These figures highlight a stronger dependence on variations in ϕ than in θ, with a clear symmetry around $\theta = \pi/2$. In Fig. S1(d), the electric potential exhibits a behavior similar to that of the product concentration and temperature; however, near the boundary between the catalytic and non-catalytic regions, the variation in ϕ is smoother compared to the previous cases.

The surface excess energy for the previous considerations is then expressed as

$$\begin{aligned} E_s^{(e)} =& A\varepsilon(\Delta H_r + \Delta\mu_r^0) \int_0^\pi \frac{\partial C_M}{\partial \phi} \sin\phi d\phi \\ &+ A\varepsilon R_g \sum_i \int_0^\pi T \ln C_i \frac{\partial C_i}{\partial \phi} \sin\phi d\phi \\ &+ FA\varepsilon \sum_i z_i \int_0^\pi \psi \frac{\partial C_i}{\partial \phi} \sin\phi d\phi \\ &- A \sum_i \gamma_{C_i} \int_0^\pi \frac{\partial C_i}{\partial \phi} \sin\phi d\phi \\ &- A\gamma_T \int_0^\pi \frac{\partial T}{\partial \phi} \sin\phi d\phi \end{aligned} \tag{S.14}$$

in which it was assumed a diluted substrate, and where ε is the interface thickness.

E: Entropy production at the interface

The entropy production rate at the particle surface is given by[5–8]

$$\begin{aligned} \sigma =& -\frac{1}{T}\mathbf{\Pi}_s : \nabla_s \mathbf{v}_s - \frac{1}{T}\mathbf{J}_M \cdot \nabla_s \mu_M - \frac{1}{T}\mathbf{J}_N \cdot \nabla_s \mu_N \\ &- \frac{1}{T^2}\mathbf{J}_q \cdot \nabla_s T - \frac{1}{T}\int_\nu J_r \frac{\partial \mu}{\partial \nu} d\nu \end{aligned} \tag{S.15}$$

in which the dissipative forces are the surface gradient of the slip velocity, $\nabla_s \mathbf{v}_s$,[5] chemical potential surface gradients of substrate, $\nabla_s \mu_M$, and product, $\nabla_s \mu_N$, temperature surface gradient, $\nabla_s T$[6,7] and $\frac{\partial \mu}{\partial \nu}$ the derivative of the chemical potential μ of the substrate along the reaction coordinate ν.[8] Furthermore, entropy production depends on the diffusive fluxes of the substrate ($\mathbf{J}_M$) and product

($\mathbf{J}_N$) currents, the heat flux ($\mathbf{J}_q$) and reactive flux in ν-space (J_r).

The mass fluxes were previously defined in Eq. (S.10), while the heat flux at the interface is approximated by $\mathbf{J}_q = \kappa \nabla_s T$, where κ is the interfacial thermal conductivity. The local contribution to the entropy production due to the chemical reaction is estimated as $\frac{R_g}{C_0} k_r C_M^2$.[8] The surface velocity corresponds to the phoretic slip velocity, given by $\xi_t \mathbf{v}_s = \int_{S'} \nabla_s \gamma dS'$, where ξ_t is a mobility coefficient and γ the surface tension.[9] The surface stress tensor for a Newtonian interface is expressed as:[5,10]

$$\mathbf{\Pi}_s = \eta_s \left(\nabla_s \mathbf{v}_s + (\nabla_s \mathbf{v}_s)^\top \right) + (\eta_d - \eta_s)(\nabla_s \cdot \mathbf{v}_s)\,\mathbf{I}_s, \tag{S.16}$$

where η_s and η_d are the surface shear and dilatational viscosities, respectively, and $\mathbf{I}_s$ is the identity tensor projected onto the surface.

Acknowledgement

The authors are grateful for the financial support of MICIU (Spanish Government) under grant No. PID2021-126570NB-I00.

Data Availability Statement

Data available in the manuscript or supplementary material. The data that support the findings of this study are available within the article.

References

(1) Debye, P. *Polar Molecules*; Chemical Catalog Company, 1929; Introduces exponential orientational relaxation in rotational diffusion.

(2) Chandler, D. Translational and rotational diffusion in liquids. I. Translational single-particle correlation functions. *The Journal of Chemical Physics* **1974**, *60*, 3500–3507.

(3) Chandler, D. Translational and rotational diffusion in liquids. II. Orientational single-particle correlation functions. *The Journal of Chemical Physics* **1974**, *60*, 3508–3512.

(4) De Groot, S. R.; Mazur, P. *Non-equilibrium thermodynamics*; Courier Corporation, 2013.

(5) Bedeaux, D.; Albano, A.; Mazur, P. Boundary conditions and non-equilibrium thermodynamics. *Physica A: Statistical Mechanics and its Applications* **1976**, *82*, 438–462.

(6) Gaspard, P.; Kapral, R. The stochastic motion of self-thermophoretic Janus particles. *Journal of Statistical Mechanics: Theory and Experiment* **2019**, *2019*, 074001.

(7) Gaspard, P.; Kapral, R. Active Matter, Microreversibility, and Thermodynamics. *Research* **2020**, *2020*.

(8) Arango-Restrepo, A.; Rubi, J.; Barragán, D. Kinetics and energetics of chemical reactions through intermediate states. *Physica A: Statistical Mechanics and its Applications* **2018**, *509*, 86–96.

(9) Arango-Restrepo, A.; Rubi, J. M. Interplay of phoresis and self-phoresis in active particles: Transport properties, phoretic, and self-phoretic coefficients. *The Journal of Chemical Physics* **2024**, *161*, 054906.

(10) Scriven, L. Dynamics of a fluid interface Equation of motion for Newtonian surface fluids. *Chemical Engineering Science* **1960**, *12*, 98–108.
